# 

**Published:** 1886-11-27

**Authors:** 


### The Hospital.

An Institution, Family, and Parochial Journal of Hospitals, Asylums, and all Agencies for the Care of the Sick, Criticism and News.

SATURDAY, NOVEMBER 27TH, 1886.

### SPECIAL NOTICE.

The Word Puzzles and "Children's Corner" will be found on p. vi of the current number.

### THE QUILTER PRIZES.

Mr. W. Cuthbert Quilter, M.P., has offered £50 in prizes, with the view of encouraging all engaged in hospital and asylum work to promote efficiency and economy in the management of these institutions. The object of these Prizes, and a full description, will be found set forth on page 115 of The Hospital of the 13th inst., with the sections and subjects suggested. The following are the conditions and the subject of the first competition:—

The Conditions.

1. We shall offer two Prizes of varying amounts, according to the importance attached to the subject of the competition. A cheque will be sent on the day the paper is published in which the results of each competition are given.

2. In all cases the names and addresses of the competitors must be forwarded, even when a *nom de plume* is used.

3. The answers must be addressed to "The Prizes Editor, The Hospital, Norfolk House, Norfolk Street, Strand," not later than the 15th December next, at noon. All communications must be written on one side of the paper only.

1. A Preliminary Competition.

At the outset we ask for suggestions from each of the classes of readers mentioned on page 115, as to the subjects in each section which they themselves consider to be the best to select for the purposes of this competition. The suggestion, under any of the heads specified, which seems to us the most practical and in all respects the best will receive the first prize of £3, and the next best suggestion the second prize of £2.

### THE LITERARY PRIZE.

A PRIZE of **One Guinea** will be given every fortnight for the best story or incident based upon hospital, or asylum, or student life, or any incident connected with the professions of medicine or nursing. Contributions may relate to any subject mentioned in the Editorial Address published on pp. 1 and 2 of the No. I issue of THE HOSPITAL, being the number for 2nd October 1886. The Prize contributions will be published in the first and third numbers of every month, and the prizes duly forwarded to the successful candidates.

### CONDITIONS.

Competitions must be sent in accompanied by the Literary Prize Coupon printed in the advertisement columns of this issue, so as to arrive at the office of THE HOSPITAL, Norfolk House, Norfolk Street, Strand, London, W.C., not later than the second and fourth Mondays in each month. Any arriving after these dates will be placed in the following fortnight's competition.

Written competitions must be on one side of the paper only. Printed matter may be sent, and is equally eligible for the Prize. Conciseness, point, and interest will be taken into account in adjudicating the Prizes in these competitions, and any competitor may send in any number of contributions for each competition. The Editor reserves to himself the right to publish any contribution sent in for competition whether successful or not. In the case of printed contributions, the source from which the communication is taken must be stated. Each communication must have the correct name and address of the sender plainly written on the first page, for publication when successful. The omission of these particulars will disqualify. Every contribution must be enclosed in an envelope and have the words "Literary Prize" plainly written in the left-hand corner.

**Result.—Next Competition.**

After due consideration of the various contributions submitted in competition for this Prize, the judges have unanimously awarded it to "Lorna Bridge" (Miss Bainbridge, Jersey), for her original story, "Poisoned: who did it?" which will be published in due course. We shall hope to hear from Lorna Bridge again; as her story is decidedly striking, and gives evidence of considerable merit in its author. A cheque for one guinea has been sent to the winner. Those who intend to compete for the current Literary Prize are reminded that the MS. must reach the Editor, Norfolk House, Norfolk Street, W.C., on or before December 13th, 1886.

### NOTICE TO CORRESPONDENTS.

All correspondence must be written on one side of the paper only.
We cannot undertake to return MSS. not used.

**Communications**, whether intended for insertion or for private information, must be authenticated by the names and addresses of the writers, not necessarily for publication.

**Local papers** containing reports or news paragraphs **should be marked.**

It is especially requested that early intelligence of **local events of interest**, or which it may be thought desirable to bring under notice, may be sent direct to the Editor.

Communications respecting editorial matters should be addressed to the Editor, Norfolk House, Norfolk Street, Strand, London, W.C.

### BUSINESS COMMUNICATIONS AND ADVERTISEMENTS.

All communications concerning business matters, non-delivery of THE HOSPITAL, etc., should be addressed to the Manager, at the Publishing Offices, 30 and 32, Sardinia Street, Lincoln's Inn Fields, London, W.C.

**Cheap Advertisements** of the "Wanted" class, including those for situations, of Twenty-four Words or under, are charged 1*s.* an insertion, or three insertions for 2*s.* 6*d.*, but must be paid for in advance. Each additional line of Eight Words is 4*d.*

**Employers requiring assistants** are charged for one insertion of Twenty-four Words or under, 2*s.* 6*d.*, or 6*s.* for three insertions. Each additional line of Eight Words is 1*s.*

**Advertisements** for insertion in the weekly issue of THE HOSPITAL **must be delivered** at the Publishing Offices **not later than 11 a.m. on Wednesdays.**

### TERMS OF SUBSCRIPTION.

Subscribers to THE HOSPITAL residing in London and the Suburbs will receive their Copies by the first post on FRIDAY MORNING. Copies for Country Subscribers are forwarded by the DAY Mail on Fridays. Terms, including postage, payable in advance:

| | Weekly Parts. | Monthly Parts. |
|---|---|---|
| Twelve months | 0 6 6 | 0 7 6 |
| Six „ | 0 3 3 | 0 3 9 |
| Three „ | 0 1 8 | 0 1 11 |

Post Office Orders should be made payable at the General Post Office, London, E.C., to WHITING AND Co., 30 and 32, Sardinia Street, Lincoln's Inn Fields, W.C. Cheques to be crossed "London and Joint Stock Bank, Limited," Chancery Lane Branch.

**Cases for Binding** the weekly or monthly parts of THE HOSPITAL in yearly vols. may be ordered of the Publishers.

### The In- and Out-patients' Hospital Diary.

(*Continued from p.* 138.)

This Diary is so arranged as to make some portion of it appear every week, and the whole in each monthly part. It has been very difficult to compile, and corrections will be very gratefully received at all times. It has been suggested that similar information about the larger Provincial and Scotch Hospitals would be useful to many readers. We should be glad to hear if this is felt to be the case.

### DISEASES AND IRREGULARITIES OF THE TEETH.—*Continued.*

| Hospitals and Terms of Admission. | Physicians, Surgeons, and Medical Officers, with Days and Hours of Attendance. |
|---|---|
| New Hospital for Women, Marylebone Road | *Surgeon*, Mr. H. Apperley |
| North-West London Hospital. See p. 16 | *Surgeon*, Mr. W. A. Maggs, F. 9 |
| Paddington Green Children's Hospital. Free. See p. 32 | *Surgeon*, Mr. C. Cottrell, W. 9 |
| Royal Free Hospital, Gray's Inn Road, W.C. | *Surgeon*, Mr. H. Harris |
| Royal Hospital for Children and Women. By Governor's letter. | *Surgeon*, Mr. Whitehouse, F. 1.30 |
| St. Bartholomew's Hospital. Free. See p. 16 | *Surgeons*, Messrs. Ewbank, Tu. 9; Paterson, F. 9 |
| St. George's Hospital. See p. 16 | *Surgeon*, Mr. Winterbottom, Tu. S. 9 and 1 |
| St. Mary's Hospital. See p. 16 | *Physician*, Dr. H. Hayward, W. S. 9.30 |
| St. Thomas's Hospital. Free. See p. 16 | *Surgeon*, Mr. Truman, Tu. F. 10 |
| University College Hospital. See p. 16 | *Surgeon*, Mr. S. J. Hutchinson, W. 9.30 |
| Victoria Hospital for Children. See p. 32 | *Surgeon*, Mr. F. Fox, S. 9.0 |
| West London Hospital. See p. 16 | *Surgeon*, Mr. H. L. Albert, Tu. F. 9.30 |
| Westminster Hospital. By Governor's letter. See p. 16 | *Surgeons*, Messrs. J. Walker, M. A. Smale, W. S. 9.15 |

### THROAT DISEASES.

| | |
|---|---|
| Central London Throat and Ear Hospital. See p. 32 | M. W. Th. S. 2, Tu. F. 5 |
| Evelina Hospital for Sick Children, Southwark Bdge. Road, S.E. | *Surgeon*, Mr. R. D. Pedley |
| Great Northern Central Hospital. Free. See p. 16 | *Surgeon*, Mr. Watson, W. 2 |
| Guy's Hospital. On payment of 3*d*. | *Surgeon*, Mr. Symonds, Th. 1 |
| Hospital for Diseases of the Throat. By payment, but free to the necessitous poor | *Surgeons*, Messrs. Wokes, T. Hovell, G. Stoker, Fenton-Jones. Daily, 1.30 |
| Infirmary for Consumption, 26, Margaret Street, W. See p. 16 | Daily, 2 |
| King's College Hospital. See p. 16 | *Surgeon*, Mr. Cartwright, Tu. F. 10 |
| Metropolitan Ear and Throat Infirmary. See p. 32 | Daily, 2.30 |
| Middlesex Hospital. See p. 16 | *Surgeon*, Mr. Hensman, Tu. 9 |
| Municipal Throat and Ear Infirmary. See p. 32 | *Physicians*, Drs. G. Holmes and J. A. Hatch, M. W. F. 10 to 12 |
| North West London Hospital. See p. 16 | M. 3 |
| St. Bartholomew's Hospital. Free. See p. 16 | *Surgeon*, Mr. Butlin, F. 2.30 |
| St. George's Hospital. See p. 16 | *Surgeon*, Mr. Whipham, Th. 1.30 |
| St. Mary's Hospital. See p. 16 | *Surgeon*, Mr. A. T. Norton, M. Th. 9.30 |
| St. Thomas's Hospital. See p. 16 | *Physician*, Dr. Semon, Tu. F. 1.30 |
| University College Hospital. See p. 16 | *Physician*, Dr. Poore, Th. 1.30 |
| Westminster Hospital. See p. 16 | W., 9 |

### DISEASES OF WOMEN (OBSTETRIC CASES, ETC.).

| | |
|---|---|
| Charing Cross Hospital. See p. 16 | *Physicians*, Drs. J. W. Black, A. Routh Tu. F. 1.30 |
| Chelsea Hospital for Women, Queen's Elm, Fulham Road. For reception of gentlewomen in reduced circumstances, and respectable poor women. Payment from 10*s*. 6*d*. a week. Gratuitous treatment to poor on recommendation of a Governor | *In-Physicians*, Drs. J. H. Aveling, A. W. Edis, F. Barnes<br>*Out-Physicians*, Drs. Dickinson and Mackeron, M. 2; W. Travers and G. Harper, Tu. 2; F. Jones and J. J. Parsons, W. 2; J. Phillips and H. T. Rutherford, F. 2 |

### DISEASES OF WOMEN (OBSTETRIC CASES, ETC.)—*Continued.*

| Hospitals and Terms of Admission. | Physicians, Surgeons, and Medical Officers, with Days and Hours of Attendance. |
|---|---|
| East London Hospital for Children and Dispensary for Women. See p. 32 | *Physicians*, Drs. E. Smith, H. B. Donkin, H. R. Crocker, J. A. Coutts, D. Williams<br>*Surgeons*, Messrs. A. Cæsar, R. W. Parker, L. A. Dunn |
| German Hospital, Dalston Lane, E. | *Physician*, Dr. Rasch, M. Th. 9 |
| Great Northern Central Hospital. Free. See p. 16 | *Physician*, Dr. F. Barnes, W. 2 |
| Grosvenor Hospital for Women and Children. See p. 32 | M. Tu. Th. S. 2 to 3 |
| Guy's Hospital. Free without letter (Uterine Wards). See p. 16 | *Physicians*, Drs. Galahin, Horrocks, M. Tu. F. 1.30 |
| Hospital of St. John and St. Elizabeth, 50, Gt. Ormond Street, W.C. For females suffering from advanced or long-standing disease. No out-patients. | *Physicians*, Drs. Constable, Tegart |
| Hospital for Women, Soho Sq., W. Free to out-patients. | *In-Physicians*, Drs. Carter, W. S. 2; R. T. Smith, Tu. F. 2<br>*In-Surgeons*, Messrs. Reeves, M. 4, Th. 2; Holland, Tu. F. 9.30<br>*Out-Physicians*, Drs. Mansell-Moullin, M. Th. 11; B. Fenwick, Tu. 10; R. T. Smith, F. 10; Oliver, S. 10<br>*Out-Surgeons*, Messrs. S. Osborne, Th. 2; Holland, W. 10 |
| King's College Hospital. See p. 16 | *Physician*, Dr. Playfair, Tu. Th. S. 2 |
| London Hospital, 281, Whitechapel Road, E. | *In-Physicians*, Drs. Herman, Tu. F. 1.30<br>*Out-Physician*, Dr. Lewers, W. S. 1.30 |
| Metropolitan Free Hospital. Free. See p. 16 | *Physician*, Dr. A. Venn, W. 2 |
| Middlesex Hospital. See p. 16 | *Physician*, Dr. Edis, Tu. F. 1.30 |
| New Hospital for Women, 222, Marylebone Road. By subscriber's letter. Out-patients pay 6*d*. entrance fee, and 2*d*. each visit. All the physicians are women | *In- and Out-Physicians*, Drs. Anderson, Atkins, Marshall, de la Cherois. Daily, 1 to 3; S. 9 to 10<br>*In- and Out-Surgeons*, Messrs. Lawson, Norton, Smith, Meredith. Daily, 1 to 3; S. 9 to 10 |
| North-West London Hospital. | W. 1.30 |
| Royal Free Hospital, Gray's Inn Road. Free. See p. 16 | *In- and Out-Physician*, Dr. Hay, S. 2 |
| Royal Hospital for Children and Women. By Governor's letter and payment of 1*d*. per week | *In-Physicians*, Drs. Park, M. Th.; Gulliver, Tu. F.; Price, W. S.; Duncan, Tu. F.<br>*In-Surgeon*, Mr. W. H. A. Jacobson, M. Th.<br>*Out-Physicians*, Drs. Park, M. Th.; Dakin, W. F.; Hadden, Tu. S.; Duncan, F. Hour, 2<br>*Out-Surgeon*, Mr. Day, W. Hour, 2 |
| St. Bartholomew's Hospital. Free. See p. 16 | *Physicians*, Drs. M. Duncan, Godson, W. S. 9<br>*Surgeons*, Messrs. Jacobson, Day, M. Th. |
| St. George's Hospital. See p. 16 | *Physician*, Dr. Champneys, Th. 1.30 |
| St. Mary's Hospital. See p. 16 | *Physicians*, Drs. Meadows, M. F. 9.30; H. Jones, M. Th. 1.30 |
| St. Thomas's Hospital. Free. See p. 16 | *Physician*, Dr. Cory, W. S. 1.30 |
| Samaritan Free Hospital for Women and Children. See p. 32 | Daily, 12 to 2 |
| University College Hospital. See p. 16 | *Physicians*, Drs. G. Hewitt, J. Williams, Tu. F. 2 |
| West London Hospital. See p. 16 | *Physicians*, Drs. A. Venn, Moullin, M S. 2 |
| Westminster Hospital. See p. 16 | *Physicians*, Drs. Potter, Tu. F. 2; Grigg, Tu. F. 9 |

### THE CHILDREN'S CORNER.

We are now only four weeks from the end of our first competition, and so near are some of my little competitors together, that the prizes still hang in the balance. Everything turns upon the four weeks which yet remain, and I therefore hope to see my little friends make especially good answers. I will promise not to make the questions difficult, so I am sure Little Mary will now be happy!

### OUR PASTIMES.

**Children's Quarterly Prize Competition.**

| | |
|---|---|
| Began .. .. .. .. .. | 2nd Oct., 1886. |
| Ends .. .. .. .. .. | 25th Dec., 1886 |

PRIZES.

| | |
|---|---|
| 1st .. .. .. .. .. | Thirty Shillings |
| 2nd .. .. .. .. .. | Twenty " |
| 3rd .. .. .. .. .. | Ten " |
| 4th .. .. .. .. .. | Five " |

will be awarded to the four competitors who shall have sent in, during the thirteen weeks, the largest number of correct answers to our pastimes.

**Ninth Competition.**

No. 44. With words unnumbered I abound;
In me mankind takes great delight:
In me much learning may be found,
Though I can neither read nor write.

No. 45. Who can evolve a little French sentence from the following?

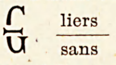

No. 46. Who can read the following sentence?
If the B m t put:
If the B . putting:

No. 47. Guess if you can, the following:
" Seogeh srev ereh wcisume vahl
Lah sehs se otreh nos llebdnas
Regni freh nos gnires rohyer
Ganoed iryd ale nifae esots sorcy
Rub nabot es rohk co caed ir."

No. 48. A miser had 56 sovereigns under the matting around his room, in the following heaps:

| | | |
|---|---|---|
| 1 | 13 | 1 |
| 13 | | 13 |
| 1 | 13 | 1 |

He found they counted 15 along each side, and determined to guard against theft by totalling up four fifteens each day. A dishonest person in the house, knowing this fact, stole eight of the sovereigns, but so arranged the money around the room that there were still fifteen sovereigns along each side. Pleased with his success, he stole yet eight more, and still left fifteen on each side as before. How did the dishonest person arrange the money? (Answers should be explained by two squares, with the necessary figures placed around.)

**Results of Seventh Competition.**

No. 32. Here I have puzzled my little readers all round, for no one has been able to make a cut in a piece of paper, measuring 4½ inches by 2 inches, so as to convert the two pieces into a square. This is how it is done:—

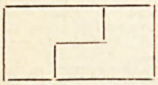

If these be placed together as follows, you get an exact square.

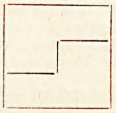

No. 33. Eleven thousand eleven hundred and eleven was easily converted into 12,111. Everybody got it right.

No. 34. The buried rivers were the Arno, Indus, Adige, Missouri, and Exe. I was surprised that Mikado did not find it out. To discover a buried town or river, you merely read the sentence over till you come across a name which is generally so arranged as to make the last syllable of one word and the first of the next; thus, "The dog always walked beh*ind us*," gives Indus, and so on with the others.

No. 35. The word of plural number is "cares." Add another "s," and the hateful foe of peace and rest turns into sweet "caress." This is a well-known conundrum.

No. 36. My first is here a "pass"; my second is a "port"; and my whole a "passport." Only Alsie and Tina have solved this enigma.

No. 37. That which is always invisible (*in* "visible") and never *out of* "sight" is surely the letter "I"!

I am very pleased with the general neatness of the answers my little correspondents send, and the evident pains which they bestow upon them. Will Florrie please to send me her full name next time she writes?

All right—None.
Five right—None.
Four right—Alsie, Bismarck, True Blue, Tina, Retsibsi.
Three right—Little Mary, Filomel, Ubique.
Two right—Mikado, Florrie, Ellie.
One right—Jimmy, T. K.

Answers, having the actual name and address (to which a pseudonym may be added for publication), must have "The Children's" Coupon attached (see advertisement pages), addressed to the "PUZZLE EDITOR," THE HOSPITAL, Norfolk House Norfolk Street, Strand W.C., not later than Thursday, 2nd December. Results will appear in our issue of the 11th December.

### "THE HOSPITAL" CHARITY BOX.

Our "Charity Box" is intended to afford a little practical help to the hospitals. Each week we propose a word for "anatomical dissection," and the first 150 competitors who extract the largest number of words from it receive the following prizes:—

| | |
|---|---|
| 1st .. .. .. | One Guinea. |
| 2nd .. .. .. | Half a Guinea. |
| 3rd .. .. .. | Five Shillings. |

The above Prizes will be repeated for every additional 150 competitors who may enter each week.

NINTH COMPETITION.

Compose the largest number of words you can from "DOCTOR."

*Observe:* Proper names, abbreviations, foreign, Latin, and obsolete words are barred.

Repetitions of similarly spelt words and of letters (unless they repeat in the word) are disallowed.

Prefixes and affixes do not count independently of words.

Write only on one side of the paper, and total your words.

Append your real name and address, adding whether Mr., Mrs., or Miss, etc.

All replies, addressed "Charity Box," THE HOSPITAL, Norfolk House, Norfolk Street, W.C., must arrive by Friday, Dec. 3rd. Results will appear Saturday, Dec. 11th.

*Every Competitor is required to cut out and enclose with his list the "Charity Box" Coupon* (see advertisement pages), *and a shilling entrance fee* (postal orders only, *crossed Lloyd's, etc., Banking Company, Limited,* THE HOSPITAL *Account.*)

The proceeds resulting from each "Charity Box" competition will be funded until the end of December 1886. The total sum available for distribution, and the names of the hospitals selected for donations, will appear on Saturday, January 8, 1887. The distribution of the proceeds will be at the Editor's sole discretion.

RESULTS OF SEVENTH COMPETITION

The "Scalpel" Competition has resulted as follows:—

| | Accepted Score. |
|---|---|
| Mr. Frank Shapley, M.R.C.S., Bryngarw, Hatherly Road, Sidcup .. .. .. .. .. | 106—1st Prize. |
| Miss A. G. Dundas, 47, Upper Brook St., W. .. | 102—2nd Prize. |
| Miss Gore, Fellenberg House, Jersey .. .. | 100—3rd Prize. |

The prizes have been forwarded to the winners.

We have specially to compliment Miss C. Hooper, Miss Hadow, and Miss Sprague on the neatness of their lists.

**Special Notice.**

In the interest of non-successful competitors, we have decided not to award the First Prize more than once to a winner in any one quarter. In the event of a competitor again making the highest accepted score, he will be placed in the second place, and if a third time highest, in the third place.

**Notices to Competitors.**

Miss Dundas.—The word set for "dissection" does not count as a word.

Mr. F. Shapley.—Words found in standard authors, if now obsolete, are not allowed.

Mrs. Wetherfield.—Prefixes and affixes cannot be regarded as "words," and we invariably bar them. We thank you for your suggestion, but the pressure upon our columns is so great that we could not spare space to print the competitions which win the first prize.

Mrs. Ellen Burton.—We fear "ipecacuanha" would be too long a word for dissection. We propose for the future to give only short words. Words of over ten letters are apt to exhaust one's patience.

Herbert Corbishley.—Prefixes and affixes are barred.

Rev. H. E. Windle.—We regret to notice the tone of your letter. The lady who took the first prize for the largest number of words out of "The Hospital" made no less than 656, of which we accepted 570. We could not occupy our space by printing the successful lists. "Repetition" applies to both letters and words. We have made this clear now. We distinctly say "foreign" words barred, and therefore the largest number of words can only mean English words. *Ergo* and *mutatis mutandis* are not English, and are therefore barred. We consider that our Rules are perfectly clear, but if there are any points about which competitors are uncertain, we are always happy to reply. We are not aware that any of our competitors have been at work "in the dark." We are sorry to hear that you do not propose to compete again. Your intimation that you shall advise others to withdraw is neither kind nor just. We can assure you that the competitions are adjudicated upon with the utmost fairness. We hope, after this explanation, that you will reconsider your decision.

